# Comprehensive transcriptome analysis of grafting onto *Artemisia scoparia* W. to affect the aphid resistance of chrysanthemum (*Chrysanthemum morifolium* T.)

**DOI:** 10.1186/s12864-019-6158-3

**Published:** 2019-10-25

**Authors:** Xue-ying Zhang, Xian-zhi Sun, Sheng Zhang, Jing-hui Yang, Fang-fang Liu, Jie Fan

**Affiliations:** College of Horticulture Science and Engineering, Shandong Agricultural University, 61 Daizong Street, Taian, 271018 China

**Keywords:** Chrysanthemum, Grafting, Aphid stress, Gene expression, RNA-Seq

## Abstract

**Background:**

Aphid (*Macrosiphoniella sanbourni*) stress drastically influences the yield and quality of chrysanthemum, and grafting has been widely used to improve tolerance to biotic and abiotic stresses. However, the effect of grafting on the resistance of chrysanthemum to aphids remains unclear. Therefore, we used the RNA-Seq platform to perform a de novo transcriptome assembly to analyze the self-rooted grafted chrysanthemum (*Chrysanthemum morifolium* T. ‘Hangbaiju’) and the grafted Artermisia-chrysanthemum (grafted onto *Artemisia scoparia* W.) transcription response to aphid stress.

**Results:**

The results showed that there were 1337 differentially expressed genes (DEGs), among which 680 were upregulated and 667 were downregulated, in the grafted Artemisia-chrysanthemum compared to the self-rooted grafted chrysanthemum. These genes were mainly involved in sucrose metabolism, the biosynthesis of secondary metabolites, the plant hormone signaling pathway and the plant-to-pathogen pathway. KEGG and GO enrichment analyses revealed the coordinated upregulation of these genes from numerous functional categories related to aphid stress responses. In addition, we determined the physiological indicators of chrysanthemum under aphid stress, and the results were consistent with the molecular sequencing results. All evidence indicated that grafting chrysanthemum onto *A. scoparia* W. upregulated aphid stress responses in chrysanthemum.

**Conclusion:**

In summary, our study presents a genome-wide transcript profile of the self-rooted grafted chrysanthemum and the grafted Artemisia-chrysanthemum and provides insights into the molecular mechanisms of *C. morifolium* T. in response to aphid infestation. These data will contribute to further studies of aphid tolerance and the exploration of new candidate genes for chrysanthemum molecular breeding.

## Background

Chrysanthemum (*Chrysanthemum morifolium* T.) is an ornamental plant with a high economic value worldwide [1, 2]; it has a long cultural history in China, where it has been used in tea and medicine, and has diverse functions, including preventing tumorigenesis and reducing blood stress [3]. However, *C. morifolium* T. is susceptible to aphids (*Macrosiphoniella sanbourni*), from seedling establishment to flowering, which reduces plant vegetative and reproductive growth [4]. In addition, severe aphid infestations can also result in serious economic losses in chrysanthemum production worldwide.

Studies have shown that grafting has been used in horticulture in China since before 2000 BC [5, 6]. Additionally, grafting is still widely used today to improve the abiotic stress tolerance of many types of plants, for example, in the cultivation of grape vines, apples, *Prunus* spp. and vegetables, by regulating many metabolic pathways and stress response processes [7, 8]. Furthermore, previous research has suggested that the herbaceous genus *Artemisia* provides a useful rootstock for enhancing abiotic stress tolerance in chrysanthemum [9–11]. However, the exact mechanisms and functions of this rootstock under biotic stress remain unclear. During grafting, a scion and rootstock are cut and adhered to each other and then undergo a series of morphological, physiological and biochemical changes [12]. The inherent genetic characteristics, tissue structures, and physiological and biochemical resistance mechanisms are intertwined, thereby enabling the grafted plants to exhibit enhanced resistance to pests and diseases [13].

Extensive efforts have been made to explicate the mechanism of aphid resistance in chrysanthemum. Numerous transcription factors have been reported to act as regulators of the response to aphid herbivory in chrysanthemum [14–16]. However, the gene expression profiles in response to aphid feeding are rarely reported in grafted chrysanthemum. During aphid infestation, a series of plant defense responses, including transcriptional regulation, plant hormone signal transduction, and the expression of different kinds of defense genes, are induced [17]. Moran [18] observed that genes associated with signaling, pathogenesis-related (PR) responses, and calcium-dependent signaling are the essential components of the aphid response profile in *Arabidopsis thaliana.* The plant hormones salicylic acid (SA), jasmonic acid (JA), and ethylene (ET) play key roles as signaling molecules during both abiotic and biotic stresses, including plant-aphid interactions [18–20]. In interactions between *Myzus persicae* and *A. thaliana*, the SA signaling pathway is activated, and the expression of genes such as *PR* genes (i.e., β-1,3-glucanase and chitinases) associated with the signaling pathway increases [21]. Plants can also activate the biosynthesis of secondary metabolites (alkaloids, terpene, phenolics, and flavonoids) that have antixenotic or antibiotic properties to deter herbivore growth, development, and reproduction [22].

In the present study, we aimed to identify the differentially regulated aphid-responsive and defense-related genes between the self-rooted grafted chrysanthemum (Cm / Cm) and grafted Artemisia-chrysanthemum (Cm / As) during aphid infestation. Our findings will enrich our knowledge of the mechanisms underlying the improvements in aphid resistance caused by grafting.

## Results

### Aphid population statistics

**Fig. 1:**
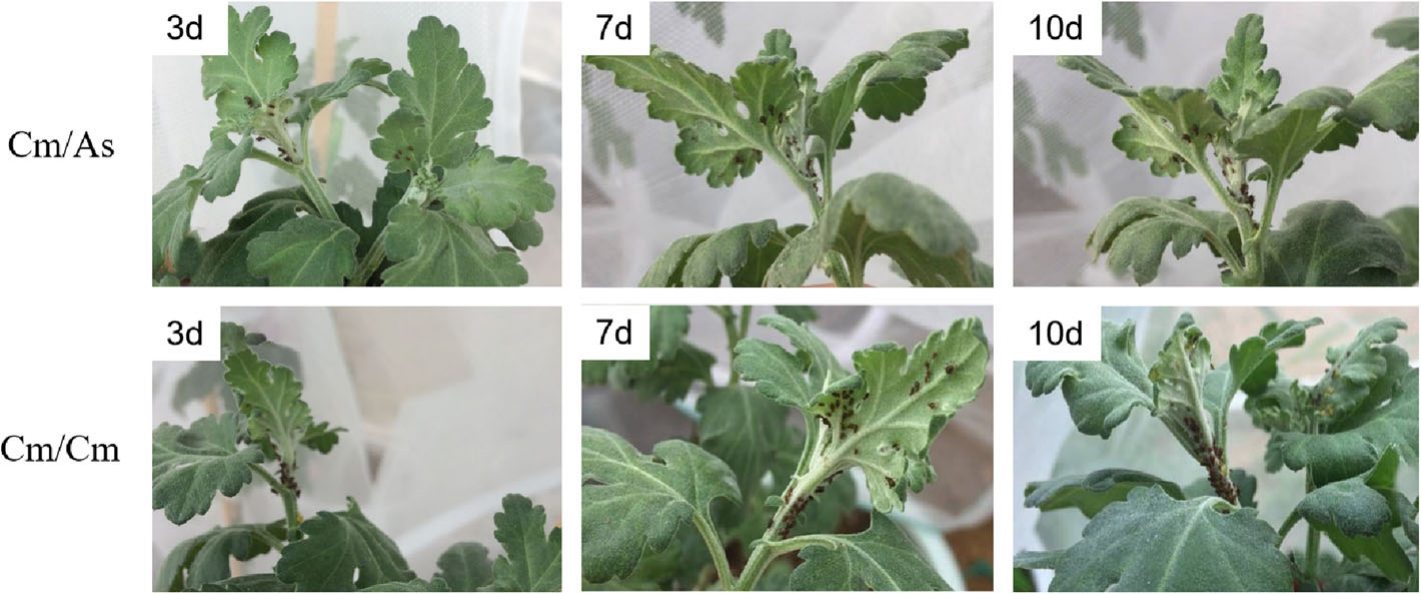
Representative pictures for aphid settlement at 3 d, 7d and 10d after aphid inoculation on selfrooted grafted chrysanthemum (Cm / Cm) and the grafted Artermisia-chrysanthemum (Cm / As)

As shown in Fig. 1 and Table 1, aphid settlement was similar in the Cm / Cm and Cm / As plants in the first 3 d after aphid infestation. On the 5th day, the aphid number between the Cm / Cm and Cm / As plants showed a significant difference (Table 1). Then, the aphids on Cm / As slowly increased, whereas the aphid number on Cm / Cm exhibited a steady and continuous increase from the 5th day to the 10th day. Thus, we hypothesized that the aphid-responsive genes that mediate defense events in the first 4 d of aphid feeding may be crucial to chrysanthemum resistance mechanisms. To unravel the molecular mechanisms involved in aphid resistance, three sampling points during a 4-d experimental period were selected for transcriptome analysis.

**Table 1:** Average number of aphids in the Cm/Cm and Cm/As

Cultivars	No. of aphids at different days after inoculation					
	1d	3d	5d	7d	9d	10d
Cm/As	23.5 ± 1.0	37.2 ± 4.3	41.4 ± 9.1^b^	42.9 ± 6.7^B^	52.9 ± 9.1^B^	52.6 ± 4.4^B^
Cm/Cm	21.6 ± 0.6	40.0 ± 4.6	50.7 ± 7.8^a^	78.9 ± 3.7^A^	100.0 ± 3.6^A^	119.8 ± 6.8^A^

Data were mean ±standard error; lowercase and capital letters represented significant differences at *p* < 0.05, *p* < 0.01

### Physiological changes in chrysanthemum under aphid stress

As shown in Fig. 5a, a higher concentration of soluble sugar was observed in Cm / As than in Cm / Cm at the same time point after infestation. Furthermore, flavonoid accumulation was observed in the leaves of Cm / As and Cm / Cm, and a higher concentration of flavonoids was observed in Cm / As than in Cm / Cm (Fig. 6b). As shown in Fig. 6c, the activity of PAL in Cm / As was significantly increased by aphid infestation and substantially higher than that in Cm / Cm. Additionally, the CAT activity in Cm / As and Cm / Cm decreased rapidly by 69.5 and 83.7% (Fig. 7b) within 6 h of infestation with aphids, and the activity of LOX (Fig. 7d) was significantly increased by aphid infestation and higher in Cm / As than in Cm / Cm.

### Transcriptome sequencing and assembly

In total, 126.66 Gb high-quality sequences were obtained from the transcriptome sequencing of the leaves of the Cm / Cm and Cm / As plants under aphid-infestation conditions, ranging from 6.25 to 8.16 Gb per sample. The average error rates of the sequences were 0.03% and more than 91% (Additional file 1: Figure S1). The assembled sequence data for these raw reads were deposited at the National Center for Biotechnology Information (NCBI) Sequence Read Archive (SRA, http://www.ncbi.nlm.nih.gov/Traces/sra) under the accession number SRP217705. The sequencing data were assembled into 147,307 transcripts with lengths ranging from 201 to 38,240 bases (mean length = 825 bases, and median length = 579 bases). As a result, 400,234 unigenes were obtained (mean length = 894 bases, and median length = 657 bases). The total length of the unigenes was 35.8 Mb (357,942,294 bases). (Additional file 1: Figure S2). All of these data indicated that the throughput was sufficiently high and the assemble quality was very better for the following analysis.

### Gene annotation and functional classification

In total, 400,232 unigenes (59.0% of the total unigenes) were annotated in the databases in this study. Most of those unigenes were annotated in the Swiss-Prot database (Table 2). Regarding to GO classification, the largest amount of annotations was in the Biological Process (BP) category, for which cellular process, metabolic process and single-organism process were the top 3 GO terms; the second largest amount of annotations was in the Cellular Component (CC) category, and the third largest amount of annotations was in the Molecular Function (MF) category. Furthermore, we compared unigenes with the KOG classification, the top 3 classes were (O) Posttranslational modification, protein turnover, chaperones, (R) General function prediction only, (J) Translation, ribosomal structure and biogenesis in this study. Based on the related biochemical pathways, the KEGG database can represent another alternative functional annotation of genes. According to the KEGG classification, the largest amount of total annotations were involved in different metabolism pathways, among which the largest pathway was carbohydrate metabolism. (Additional file 1: Figure S3).

**Table 2:** Annotation of unigenes in different database

Database	No. of annotated unigenes	Percentage of annotated unigenes (%)
NR	42,859	10.7
NT	102,642	25.64
KO	81,327	20.31
SwissProt	163,850	40.93
PFAM	161,161	40.26
GO	161,161	40.26
KOG	47,002	11.74
all Databases^a^	3747	0.93
at least one Database^b^	236,851	59.17
Total Unigenes	400,234	100

^a^Annotated in at least one of the above databases

^b^Annotated in all of the above databases

These results provide a valuable resource for investigating which genes involved in response to *M. sanbourni* infestation might play a key role in chrysanthemum molecular breeding.

### Differential expression and gene functional classification

**Fig. 2:**
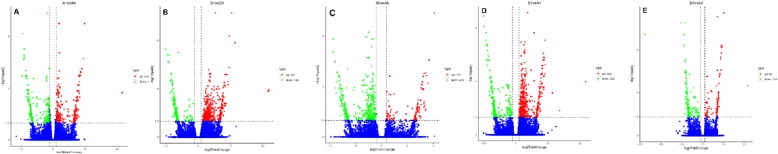
Volcano plots of differentially expressed sequences in selfrooted grafted chrysanthemum and the grafted Artermisia-chrysanthemum. **a** The up-regulated or down-regulated genes in A1 compared to A0. **b** The up-regulated or down-regulated genes in B1 compared to B0. **c** The up-regulated or down-regulated genes in B0 compared to A0. **d** The up-regulated or down-regulated genes in B1 compared to A1. **e** The up-regulated or down-regulated genes in B2 compared to B2. (The scattered points represent the genes, the blue dots represent the genes with no significant difference, the red dots represent the up-regulated genes with significant differences, and the green dots indicate the down-regulated genes with significant differences)

We compared the DEGs among the multiple groups of samples. As shown in Fig. 2b, there was a large number of DEGs in B1 compared to B0, with 457 genes upregulated and 163 genes downregulated. However, there were 219 upregulated genes and 188 downregulated genes (Fig. 2a) among the DEGs in A1 compared to A0. From the overall expression of DEGs induced under aphid stress, the number of upregulated genes was higher than that of downregulated genes. The results indicated that most of the genes in chrysanthemum leaves were activated, and few genes were inhibited after stimulation. In total, the number of different genes in Cm / As was approximately twice that in Cm / Cm.

**Fig. 3:**
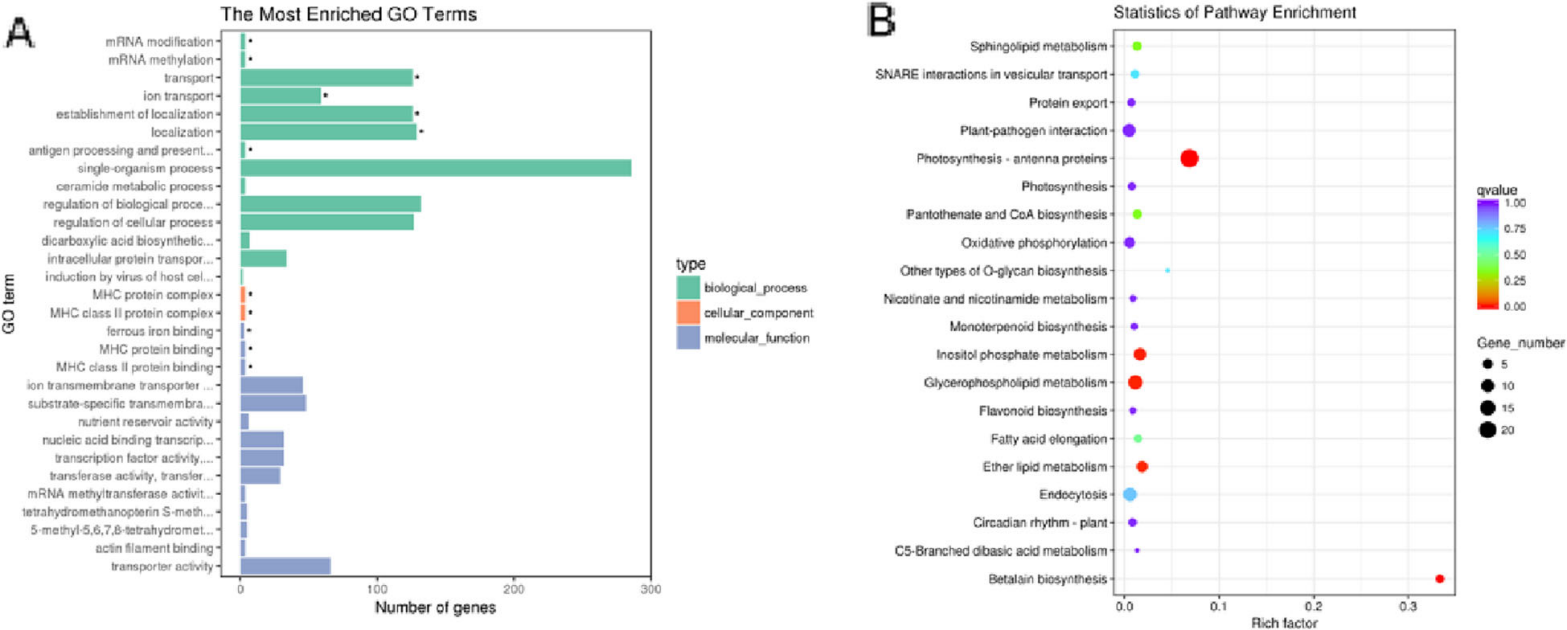
Enrichment analysis of differently expressed genes between the selfrooted grafted chrysanthemum and grafted Artermisia-chrysanthemum. **a** Functional enrichment categories of DEGs in Gene Ontology. (* indicates significant enrichment). **b** Scatterplot of enriched KEGG pathway of DEGs (Top 20)

Furthermore, the results showed that there were 1337 DEGs, among which 680 were upregulated and 667 were downregulated, in the Cm / As compared to the Cm / Cm. Among them, there were 504 genes with different expression in B0 compared to A0, of which 134 genes were upregulated and 370 genes were downregulated (Fig. 2c); 620 genes were differentially expressed in B1 compared to A1, of which 457 genes were upregulated and 163 genes were downregulated (Fig. 2d); and 223 genes were differentially expressed in B2 compared to A2, with 89 upregulated genes and 134 downregulated genes (Fig. 2e). A total of 1337 DEGs were identified, as shown in Fig. 3. According to the GO classification (Fig. 3a), the largest amount of annotations was in the BP category, for which the top 3 GO terms were single-organism process, establishment of localization and regulation of biological; the second most abundantly annotated category was MF, for which the top 3 terms were transporter activity, ion transmembrane transporter and substrate-specific transmembrane; and the third most abundantly annotated category was CC, for which the top term was MHC protein complex. The results of the KEGG pathway classification were shown in Fig. 3b, mainly including photosynthesis, trans-linolenic acid metabolism, pantothenic acid and CoA biosynthesis, plant hormone signal transduction pathways, phenylpropane biosynthesis, betaine synthesis, monogamous biosynthesis, sucrose metabolism, flavonoid biosynthesis, and plant-pathogen interactions. The results indicated that grafting chrysanthemum onto *A. scoparia* W. resulted in enhanced resistance to aphids, which was closely related to the regulation of metabolic pathways, secondary metabolic biosynthesis, immune response and signal transduction.

### Verification of DEGs using qRT-PCR

**Fig. 4:**
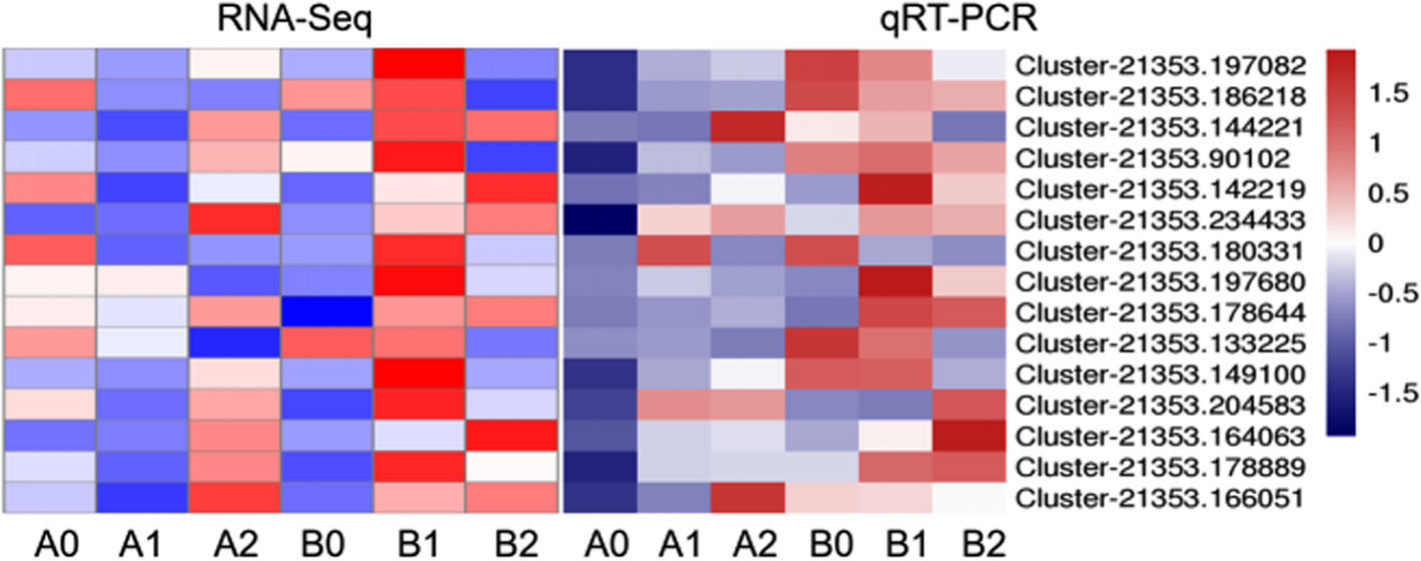
Comparisons of the expression changes of selected genes during aphid stress in qRT-PCR and RNA-Seq analysis were used in heat map analysis. Different colors indicate different levels of genes expression: from red to green, the value ranges from large to small

To validate the results of Illumina RNA-Seq, fifteen genes from the library of the grafted and self-rooted seedlings after aphid infestation were chosen randomly for qRT-PCR. For comparison, clustering heat maps between the RNA-Seq and qRT-PCR data were generated. As shown in Fig. 4, the qRT-PCR results revealed that the expression tendency of these genes showed significant similarity with the Illumina RNA-Seq data, suggesting the reproducibility and accuracy of the RNA-Seq results.

## Discussion

### Genes related to sucrose metabolism

**Fig. 5:**
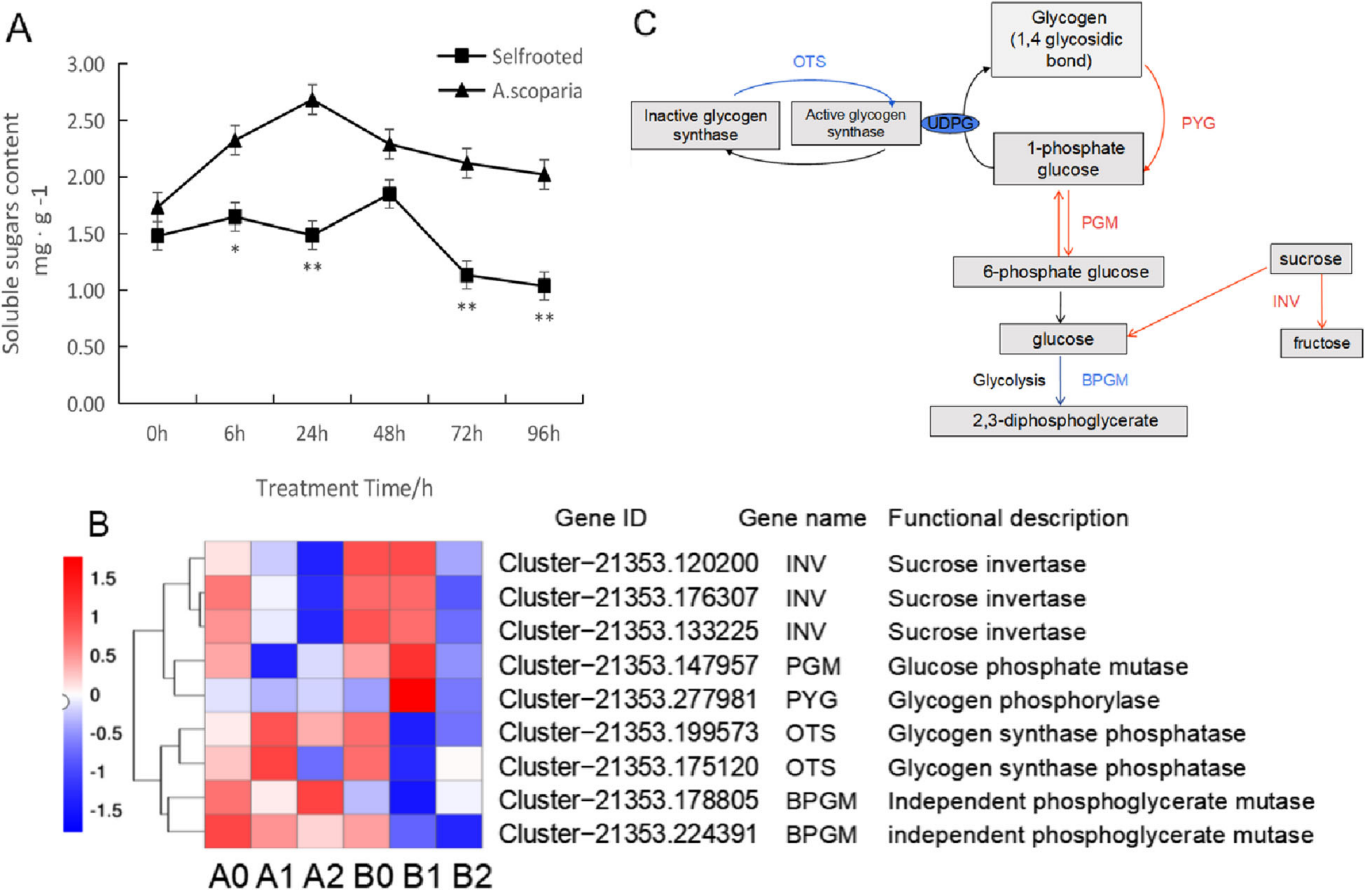
Sucrose metabolism related genes. **a** Effect of aphid stress on content of total sugars (* and ∗∗indicate significant differences (respectively, *P* ≤ 0.05 and *P* ≤ 0.01). **b** Heat map of the DEGs involved in sucrose metabolism. **c** The metabolic pathway analysis of unigenes involved in sucrose metabolism (Red and blue lines indicate up-regulated and down-regulated genes in Cm / As compared to Cm / Cm, respectively)

Sucrose has a central position in plant metabolism as the first free sugar formed during photosynthesis [23, 24] and the major form of translocated sugar in the phloem [25]. Soluble sugars, such as sucrose, glucose and fructose, can be donors of carbon skeletons for secondary metabolism and signaling molecules that regulated the expression of genes encoding flavonoid biosynthesis enzymes [26]. Sucrose metabolism plays an important role in the defense against insect herbivores, such as aphids. In our study, as shown in Fig. 5a, the level of soluble sugar increased after 6 h of infestation and reached a maximum at 24 h in the Cm / As plants. At the same time point, a higher concentration of soluble sugar was observed in Cm / As than in Cm / Cm.

The multiple roles of plant sucrose transporters, especially the central role of sucrose loading into the phloem, suggest that sucrose transport is strongly regulated by biotic stresses [27–29]. In the present study, nine genes involved in the sucrose metabolism pathway were differentially expressed between Cm / As and Cm / Cm. As shown in Fig. 5b, three *INV* genes (Cluster-21,353.120200, Cluster-21,353.176307 and Cluster-21,353.133225), one *PYG* gene (Cluster-21,353.277981) and one *PGM* gene (Cluster-21,353.147957) were strongly upregulated in Cm / As (B1) compared to Cm / Cm (A1) at 6 h, and these genes have been shown to be involved in glucose synthesis (Fig. 5c). In addition, there were four downregulated genes in B1 compared with A1, including two *OTS* genes (Cluster-21,353.199573 and Cluster-21,353.175120) and two *BPGM* genes (Cluster-21,353.178805 and Cluster-21,353.224391), which have been shown to be involved in glucolysis (Fig. 5c). The results suggested that grafting chrysanthemum onto *A. scoparia* W. accelerated the synthesis of soluble sugars, which increased the carbon metabolism burden of aphids and thus prevented aphids from feeding on the leaves. The details of these genes are shown in Additional file 2: Table S1. These genes may play an important role in promoting the synthesis of soluble sugars at the early stage in response to *M. sanbourni* infestation in the Cm / As seedlings.

### Genes related to secondary metabolism

**Fig. 6:**
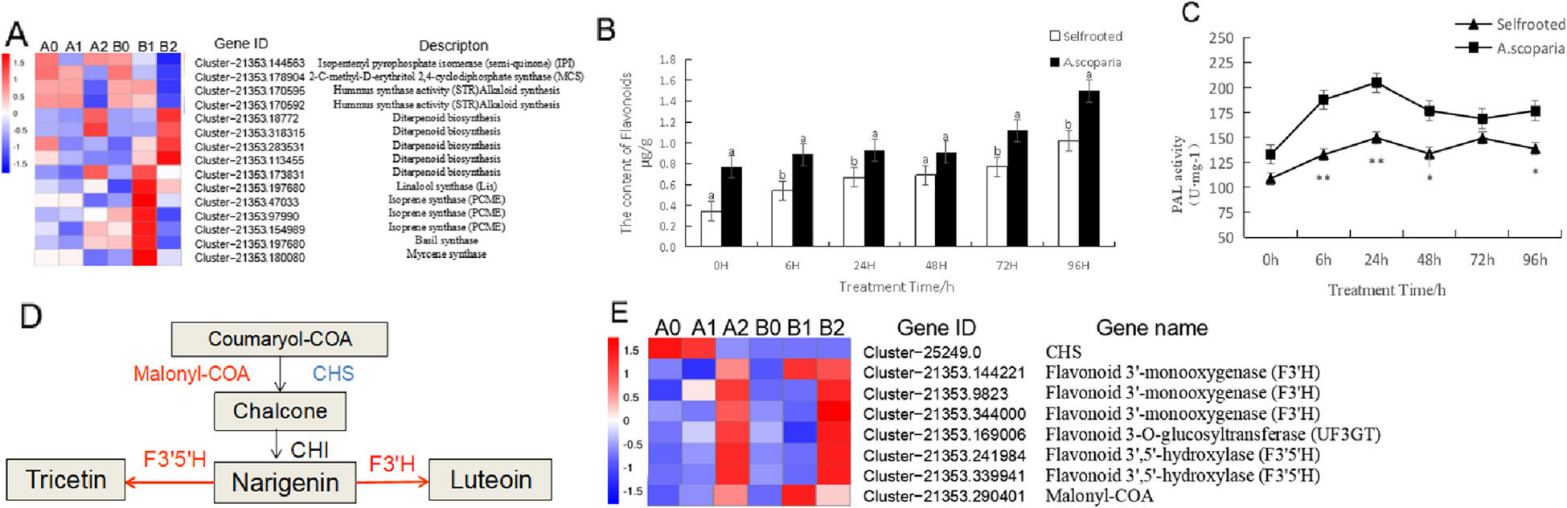
**a** Heat map of the genes involved in synthesis of terpenoids and nitrogen-containing secondary organisms. **b** Effect of aphid stress on content of flavonoids in Cm / As and Cm / Cm. **c** PAL activity in leaves of Cm / As and Cm / Cm. **d** The metabolic pathway analysis of unigenes involved in flavonoid biosynthesis of Cm / As. Red and blue lines indicate up-regulated and down-regulated genes in Cm / As compared to Cm / Cm, respectively. **e** Heat map of the genes involved in flavonoids pathway under aphid stress

Secondary metabolites, for example, flavonoids, terpenes, phenolics and alkaloids, have antixenotic or antibiotic properties and thus function in the defense against insect herbivores, such as aphids [22]. Among secondary metabolites, flavonoids are responsible for many key functions, which are critical for plant survival [30]. Flavonoids are catalyzed by amount of enzymes, such as PAL, which has been studied in plant responses to biotic and abiotic stresses [31]. In the present study (Fig. 6c), the activity of PAL in Cm / As was significantly increased by aphid infestation and higher than that in Cm / Cm, which promoted the accumulation of flavonoids. Studies on insect-plant interactions have revealed important contributions of flavonoids [32]. In Vigna [33], there was a positive relationship between resistance or susceptibility properties against aphids and the flavonoid glycoside content. The content of flavonoids in susceptible lines was lower than that in resistant lines [34]. In the present study, after *A. sanbourni* infestation, flavonoid accumulation was detected in the leaves of Cm / As and Cm / Cm. The level of flavonoids increased after 6 h of infestation, and a higher concentration of flavonoid was observed in Cm / As than in Cm / Cm (Fig. 6b). Additionally, genes related to flavonoid synthesis were also identified. Eight genes were differentially expressed between Cm / As and Cm / Cm (Fig. 6d-e), including one *CHS* gene (Cluster-25,249.0), which was downregulated in B1 compared to B0, and one *malonyl-CoA* gene (Cluster-21,353.290401), which was strongly upregulated in B1 compared to A1 and may play an important role in Cm / As defense towards aphid feeding at the early stage of infestation. In addition, three *flavonoid 3′-monooxygenase* (*F3’H*) genes (Cluster-21,353.144221, Cluster-21,353.9823 and Cluster-21,353. 344,000), two *flavonoid 3′5’-hydroxylase* (*F3’5’H*) genes (Cluster-21,353.241984 and Cluster-21,353. 339,941) and one *flavonoid 3-O-glucosyltransferase* gene (Cluster-21,353.169006) were strongly upregulated in B2 compared to A2, which could play an important role in Cm / As defense towards aphid feeding in the later stage of infestation (Fig. 6d).

In addition, among secondary metabolites, alkaloids act as a defense against insects and other herbivores [35, 36]. For example, the reduced alkaloid content in sweet lupins led to a high susceptibility to insect herbivores, e.g., aphids [37–39]. In this study, only two *alkaloid synthesis-related* genes, *STR* genes (Cluster-21,353.170595 and Cluster-21,353.170592), were detected in Cm / As and Cm / Cm, both of which were downregulated at 96 h after aphid infestation.

Plants produce a vast array of volatiles that mediate their interaction with the environment and that consist of terpenoids and isoprenoids, which are synthesized through the condensation of *C5* isoprene units. Monoterpenes and sesquiterpenes represent the *C10* and *C15* terpene classes, respectively. These compound classes have typical characteristics, such as volatility, flavor/aroma, and toxicity, and hence play important roles in plant defense and pollinator attraction [40]. A previous study found that the increased content of monoterpenoids and sesquiterpenoids in the leaves of the hybrid between chrysanthemum and *Artemisia vulgaris* enhanced the resistance of the plant to aphids [9]. In the present study, we obtained several DEGs related to terpenoid synthesis (Fig. 6a). There were three genes related to monoterpene synthesis, one basil synthase gene (Cluster-21,353.197680), one myrcene synthase gene (Cluster-21,353.180080) and three isoprenoid genes (Cluster-21,353.97990, Cluster-21,353.154989 and Cluster-21,353.47033). All of these genes were strongly upregulated in B1 compared to A1, indicating their potential roles in the defense responses against aphids in the early stage after grafting on *A. scoparia* W. Furthermore, five genes involved in diterpenoid biosynthesis, including one gene (Cluster-21,353.173831), were strongly upregulated in B1 compared to A1, and four genes (Cluster-21,353.18772, Cluster-21,353.318315, Cluster-21,353.283531 and Cluster-21,353.113455) were strongly upregulated in B2 compared to A2 and may play important roles in responding to aphids in the later stage of infestation.

The details of these genes discussed above are shown in Additional file 3: Table S2, which illustrates the involvement of secondary metabolites during aphid herbivory in Cm / As and Cm / Cm leaves, indicating that using the *A. scoparia* W. rootstock could alleviate aphid stress in chrysanthemums by altering these gene profiles, which play potential roles in defense responses to aphids.

### Plant hormone signaling pathway involved in plant-aphid interaction

**Fig. 7:**
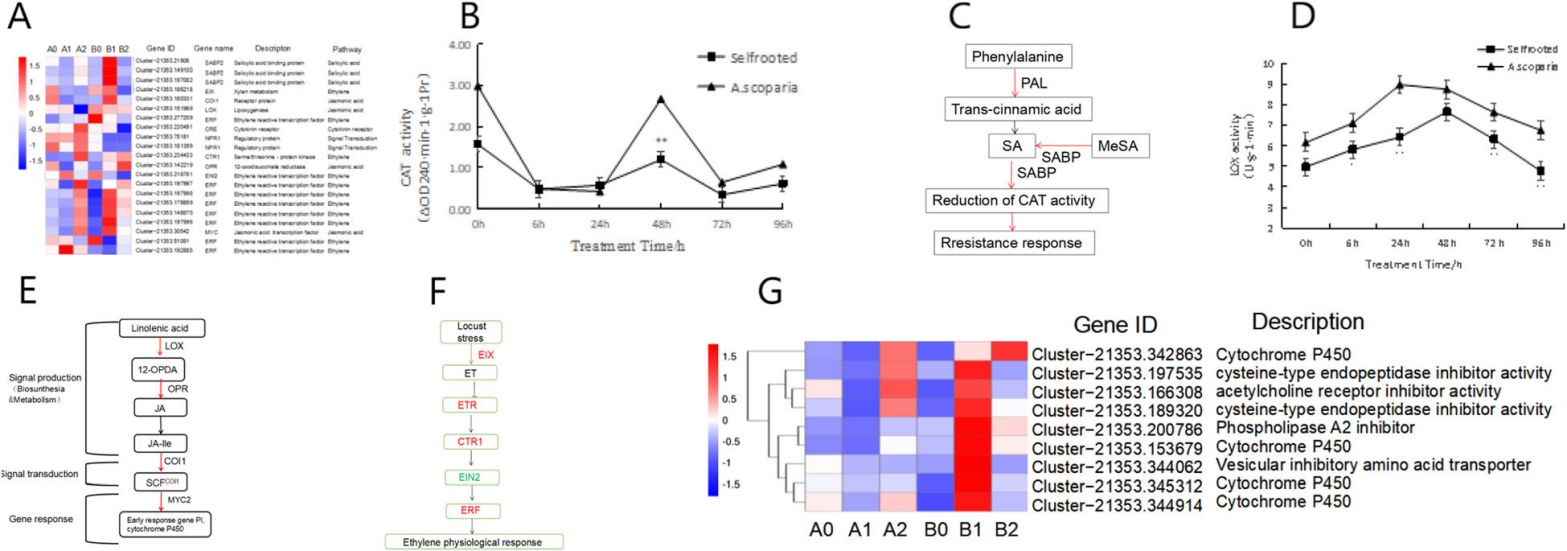
Plant hormone signal transduction between Cm / As and Cm / Cm. **a** Heat map of the DEGs involved in plant hormone signal transduction between Cm / As and Cm / Cm under aphid stress. **b** CAT activity in leaves of Cm / As and Cm / Cm. **c** The metabolic pathway analysis of unigenes involved in SA of Cm / As. **d** LOX activity in leaves of Cm / As and Cm / Cm. **e** The metabolic pathway analysis of unigenes involved in JA biosynthesis of Cm / As. **f** The metabolic pathway analysis of unigenes involved in ET biosynthesis of Cm / As. Red and blue lines indicate up-regulated and down-regulated genes in Cm / As compared to Cm / Cm, respectively. **g** Heat map of the DEGs involved in down stream response genes

JA, SA and ET are three major phytohormones involved in the regulation of signaling networks related to aphid-infestation defense responses. As shown in Fig. 7a, hormone transduction pathway-related genes were expressed at different time points between Cm / As and Cm / Cm. A total of 21 DEGs were involved in several plant hormone signal transduction pathways, including cytokinins (CKs), ET, JA and SA, in this study. The details of these genes discussed above are shown in Additional file 4: Table S3.

The JA pathway and SA pathway are two main pathways involved in plant-induced defense [41]. Phytohormonal crosstalk between JA- and SA-mediated signaling pathways is thought to underlie plant-mediated interactions among multiple insect species and the behavioral responses of parasitoids and predators [10, 42, 43]. The activation of SA signaling in response to aphid feeding [18, 44, 45] may suppress JA-dependent indirect defense responses. In our study (Fig. 7a), one *LOX* gene, which is a key enzyme in JA synthesis, was downregulated in B1 compared to B0, whereas the *SABP* genes, which are key enzymes in SA synthesis, were strongly upregulated in B1 compared to B0.

Exogenous SA treatment can enhance plant resistance to pathogenic bacteria and induce the expression of disease-related genes at the same time [46, 47]. SABP2 catalyzes the conversion of MeSA into SA, which is essential for inducing tobacco systemic acquired resistance (SAR) [48]. In this study, three *SABP2* genes (Cluster-21,353.21806, Cluster-21,353.149100 and Cluster-21,353.197082) were upregulated by aphid infestation in B1 compared to A1 in the early stage. In addition, within 6 h of infestation with aphids, the CAT activity in the Cm / As and Cm / Cm plants decreased rapidly by 69.5 and 83.7%, respectively (Fig. 7b). This result might be due to the change in the conformation of CAT, which binds to the *SA-binding protein* (*SABP1*) [49], inhibiting the activity of CAT and activating the expression of disease course-related protein genes. We suggest that grafting chrysanthemum onto *A. scoparia* W. inhibited the activity of CAT through the upregulation of *SABP2* genes after aphid infestation (Fig. 7c), and then the self-feedback mechanism was initiated, which amplified the signal transduction in the cell and ultimately induced the expression of the disease-related protein genes (Fig. 7g). Furthermore, in the SA-mediated SAR reaction of *Arabidopsis*, *NPR1* is a crucial regulatory protein that impacts the SAR pathway downstream of the SA signaling pathway. During SAR, *NPR1* induces *PR* gene expression, and even after induction by SA, BTH or INA, *NPR1* expression levels increase [50, 51]. However, in this study (Fig. 7a), two *NPR1* genes were downregulated in B1VSA1, suggesting that the *NPR1* gene had different regulatory mechanisms on aphid stress after grafting onto *A. scoparia* W*.*

JA affects not only the growth of many plants but also the resistance of plants to pathogen infection. LOX and oxo-phytodienoic reductase (OPR) are not only key enzymes for JA synthesis but also important substances that induce defense mechanisms in plants [52–54]. As the receptor in the JA signaling pathway, *COI1* (*COR-insensitive1*) can interact with *SKP1* (*S-phase kinase-associated protein1*) [55]. *LOX* genes were significantly upregulated by *M. persicae* feeding on *A. thaliana* leaves [21], *M. nicotianae* feeding on *Nicotiana attenuata* leaves [56], and *M. euphorbiae* feeding on tomato leaf tissues [57]. In our study (Fig. 7e), the overall expression level of one *LOX* gene (Cluster-21,353.151969) in the Cm / As plants was higher than that in the Cm / Cm plants at the same time point. Additionally, the activity of LOX (Fig. 7d) in Cm / As was significantly increased by aphid infestation and higher than that in Cm / Cm, which promoted the accumulation of JA. Compared with A2, B2 exhibited one *OPR* gene (Cluster-21,353.142219) that was strongly upregulated. In addition, one *COI1* gene (Cluster-21,353.180331) and one *MYC* gene (Cluster-21,353.197996) were strongly upregulated in B1 compared to A1 and responded rapidly to aphid stress in the early stage of infestation. In addition, *protease inhibitors* (*PIs*) are widely found in organisms, regulate many important biological activities in plants and have a protective effect against insects [58]. These genes above all activated the expression of cytochrome P450 genes and protease inhibitor genes, which were downstream response genes (Fig. 7g) responding to aphid stress. The details of these genes discussed above are shown in Additional file 5: Table S4. In this study, four *cytochrome P450* genes (Cluster-21,353.342863, Cluster-21,353.153679, Cluster-21,353.345312 and Cluster-21,353.344914) were upregulated by aphid infestation in B1 compared to A1. Protease inhibitors were divided into four categories, *serine protease inhibitors*, *metal carboxypeptidase protease inhibitors*, *cysteine protease inhibitors* and *aspartic acid protease inhibitors*, according to the active sites of the active enzymes [59]. In our study, two *cysteine protease inhibitor* genes (Cluster-21,353.197535 and Cluster-21,353.189320) and one *phospholipase A2 inhibitor* gene (Cluster-21,353.200786) were significantly upregulated by aphid infestation in B1 compared to A1. As important enzymes, the acetylcholine receptor inhibitor gene and the *phospholipase A2 inhibitor* gene can inhibit the nerve conduction of insects, which may play an important role in responding to aphids in the early stage of infestation after grafting onto *A. scoparia* W.

Some studies have proven that aphid infestation markedly increases the production of ET in the leaves of plants [60–62]. *Ethylene-inducing xylanase* (*EIX*) induces ET production and is an effective elicitor of plant defense responses in specific cultivated species of *Nicotiana tabacum* and *Lycopersicon esculentum*. In the present study, one *EIX* gene (Cluster-21,353.186218) was strongly upregulated in B1 compared to A1, which may stimulate the accumulation of ET (Fig. 7f). *CTR1* is a negative regulatory component downstream of the ET receptor and the first cloned gene in the ET signal pathway [63, 64]. In this study (Fig. 7a), one *CTR* gene (Cluster-21,353.234433) was upregulated in B1 compared to A1 at an early time point after aphid infestation. Research shows that *EIN2* has important effects in the ET signal pathway, and the loss of *EIN2* gene function leaves the plant completely insensitive to ET [65]. In this study (Fig. 7a), one *EIN2* transcription factor gene was downregulated in B1 compared to B0, but it was highly expressed in A1. *ET-responsive factor* (*ERF*) encodes a transcriptional activator to promote several downstream ET-responsive genes. Additionally, *MYC* and *ERF* transcription factors, participate in the ET and JA signaling pathways and activate defense-related genes. In our study, nine *ERF* genes were involved; among them, four genes (Cluster-21,353.197997, Cluster-21,353.197998, Cluster-21,353.176859 and Cluster-21,353.148870) were strongly upregulated in B1 compared with A1 (Fig. 7a).

These DEGs were identified in the comparison of Cm / As and Cm / Cm, as discussed above, which illustrates the involvement of the plant hormone signaling pathway during aphid herbivory, indicating their potential roles and the complex connections in the defense responses against aphids after grafting chrysanthemum onto *A. scoparia* W.

### Plant-pathogen interaction

**Fig. 8:**
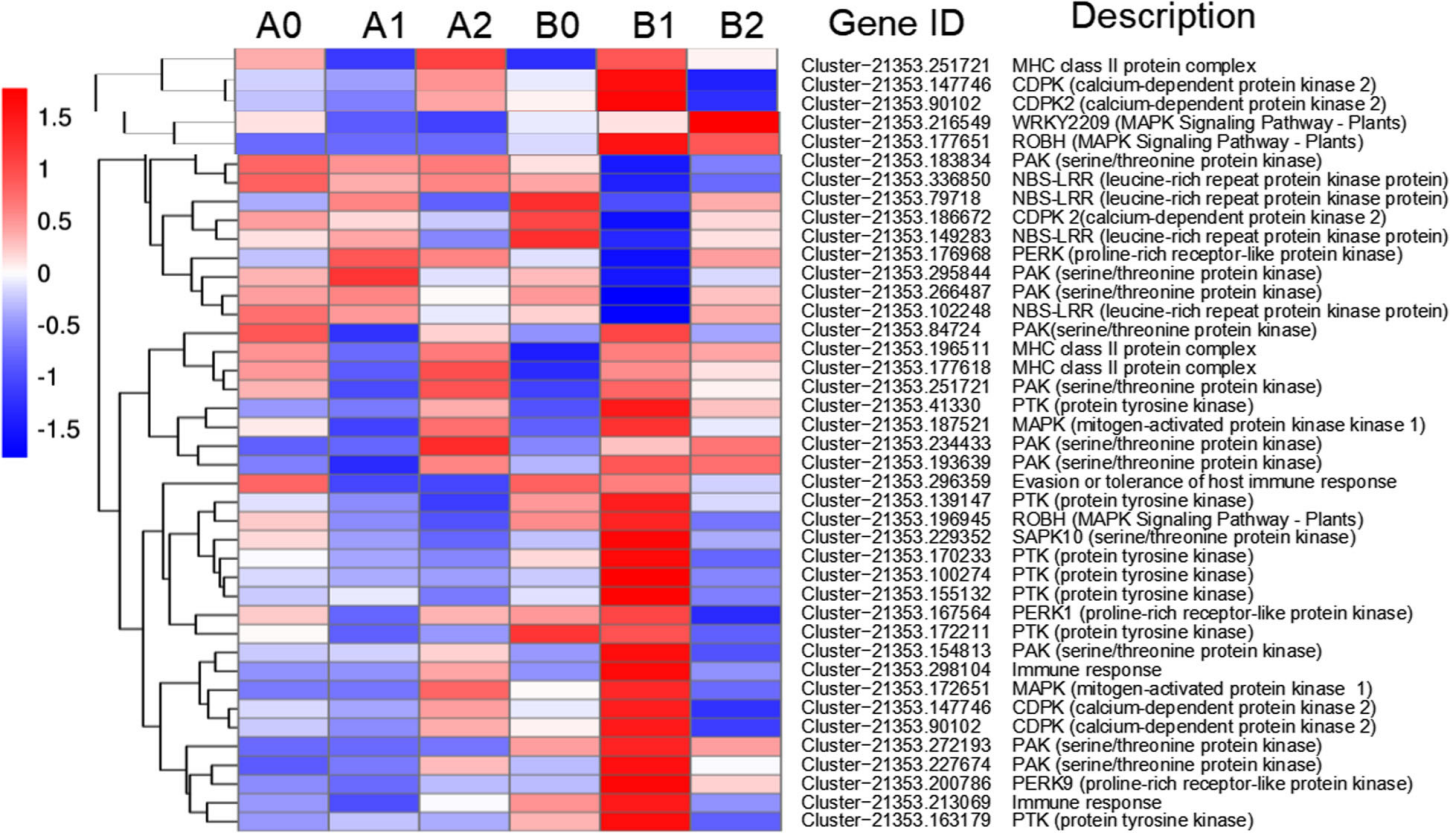
Heat map of the DEGs involved in plant-pathogen interaction

Two layers of plant immunity are well defined: pattern-triggered immunity (PTI) and effector-triggered immunity (ETI) [66]. As the first line of the innate immune response, PTI is triggered by the perception of herbivore-associated molecular patterns (HAMPs) in the case of herbivory and microbe-associated molecular patterns (MAMPs) or pathogen-associated molecular patterns (PAMPs) by cognate plasma membrane-localized pattern recognition receptors (PRRs) in the case of microbial infection [67]. The downstream immune signaling pathways are activated by different PRRs, including the receptor of kinases and the activation of various kinases, such as mitogen-activated protein kinase (MAPK) and calcium-dependent protein kinase (CDPK) [68]. In this study, four genes in the MAPK signaling pathway were recognized (Fig. 8): one *ROBH* gene (Cluster-21,353.196945) and one *MAPK* gene (Cluster-21,353.172651), which were upregulated in B1 compared to A1, and one *WRKY2209* gene (Cluster-21,353.216549) and one *ROBH* gene (Cluster-21,353.177651), which were upregulated in B2 compared to A2.

Ca^2+^ regulates many important physiological processes. Transcriptome and metabolome changes in *Arabidopsis* were surveyed at 6 h, 12 h, 24 h and 48 h after *B. brassicae* infestation, revealing that reactive calcium is involved in early signaling [69]. Plant defenses against phloem-feeding insects involve multiple signaling cascades, and molecular genetic studies on the model plant *A. thaliana* have demonstrated that *leucine-rich repeat* (*LRR*) family receptor-like kinases and calcium signaling proteins are partially activated by phloem feeding [70]. MHCII molecules can mediate the transmission of reverse signals and influence and regulate many physiological processes in immune cells [71]. Studies have shown that *CDPK2* and *MHCII* can regulate each other in two major antigen presenting cells [60]. In the present study (Fig. 8), four *CDPK* genes (Cluster-21,353.147746, Cluster-21,353.90102, Cluster-21,353.147746 and Cluster-21,353.90102) and three *MHCII* genes (Cluster-21,353.251721, Cluster-21,353.196511 and Cluster-21,353.177618) were specifically upregulated in B1 compared to A1, which may play an important role in the early stage of responding to *M. sanbourni* infestation in Cm / As seedlings.

Two cloned aphid resistance genes, *Mi-1.2*, confer resistance to the potato aphid, *Macrosiphum euphorbiae* (Thomas) [72, 73], and Vat mediates resistance to the cotton aphid *Aphis gossypii* Glover [74, 75], all of which belong to the NBS-LRR family. Similarly, we found four such DEGs (Cluster-21,353.336850, Cluster-21,353.79718, Cluster-21,353.149283 and Cluster-21,353.102248) between the Cm / Cm plants and the Cm / As plants, but these DEGs require further cloning and functional identification to confirm the presence of the NBS-LRR region.

Furthermore, in our study (Fig. 8), there were many DEGs related to tyrosine protein kinases and serine/threonine protein kinases in the interaction between the plants of the Cm / Cm and the Cm / As. Eight *PAK* genes and six *PTK* genes were specifically upregulated in B1 compared to A1. These protein kinase genes may be closely related to grafting to improve the aphid resistance of *C. morifolium* T. (The details of these genes discussed above are shown in Additional file 6: Table S5).

## Conclusions

This study showed that the number of aphids was significantly lower in Cm / As than in Cm / Cm 10 d after aphid infestation. The results indicated that grafting chrysanthemum onto *A. scoparia* W. resulted in enhanced resistance to aphids. In addition, the content of soluble sugars and the accumulation of flavonoids in Cm / As were higher than those of Cm / Cm at the same time point under aphid stress. Transcriptome results showed that grafting onto *A. scoparia* W. affected aphid-responsive and defense-related gene expression after feeding, mainly in the processes of sucrose metabolism, plant hormone signaling, secondary metabolism and plant-pathogen interaction. Intercommunication typically occurred between the different defense pathways to which these genes belonged, thereby providing the Cm / As seedlings the ability to integrate environmental, developmental and defense-related signals and to finetune their defense responses to aphids.

## Methods

### Study sites and sampling

#### Plant materials

The cultivar *Chrysanthemum morifolium* T. ‘Hangbaiju’ is a national geographical indication product in Tongxiang, Zhejiang Province, China. The material has been deposited in Chinese Virtual Herbarium, and the deposition number is JJF00030415. The materials were collected in 2010 in Tongxiang, authenticated by Professor Chengshu Zheng (Shandong Agricultural University) and then preserved in the Chrysanthemum Germplasm Resource Preservation Center at Shandong Agricultural University. In the present study, ‘Hangbaiju’ was obtained from the Chrysanthemum Germplasm Resource Preservation Center, and plants were subsequently verified by Shandong Agricultural University scientists based on Cheng-shu Zheng. The seeds of *Artemisia scoparia* W. was obtained from the Chrysanthemum Germplasm Resource Preservation Center which were sown on March 10th in plastic pots (19 cm in diameter × 17 cm in depth) filled with a 2:1:1 (v/v/v) mixture of garden soil, peat and vermiculite. On May 1st, healthy *A. scoparia* W. and chrysanthemum plants 30 cm in height and with a 6-mm stem diameter were used as rootstocks, and apical shoots with a length of 14~16 cm and a stem diameter of 4 mm from healthy chrysanthemum were used as scions. Insert grafting was performed as described by Lee (Lee 1994). On July 9th, morphologically uniform Cm / Cm and Cm / As plants were selected for the aphid infestation treatment. The plants were arranged in a fully randomized manner, and the experiment was repeated three times, with each replicate consisting of 15 plants.

#### Aphid infestation

Aphids (*M. sanbourni*) were collected from field-grown chrysanthemum plants, and second-instar nymphs were fostered and used to inoculate the plants. The third fully expanded leaves from the tip of the stem of the Cm / Cm and Cm / As plants were infested with twenty aphids (4 h of starvation) transferred by a soft brush. Twenty plants per treatment were repeated three times. The infested plants were caged with a transparent ventilated plastic net. Aphids were counted 1 d, 3 d, 5 d, 7 d, 9 d and 10 d after aphid inoculation.

The third fully expanded leaves of seedlings for each treatment were harvested at 0 h, 6 h, and 96 h. Before harvest, aphids were removed with a soft brush, which enabled the aphids to be removed from the leaves by flushing the plants with deionized water. Harvested materials were immediately frozen in liquid nitrogen and stored at − 80 °C for the following experiments. The samples collected at defined time points of each treatment were pooled for RNA-Seq.

### RNA extraction and transcriptome sequencing

The RNA isolation was performed using the CTAB method according to Chang et al. [76]. A total amount of 1.5 μg RNA per sample was used as input material for the RNA sample preparations. Sequencing libraries were generated using the NEB Next® Ultra™ RNA Library Prep Kit for Illumina®. RNA from three leaves of the same plant were pooled equimolarly and used as a single sample for the following transcriptome sequencing. Finally, these libraries were sequenced at Novogene Biological Information Technology Co., LTD. (Tianjin, China) using the Illumina Hiseq 4000 platform (San Diego, CA, USA). The transcriptome of each treatment was analyzed at three time points (0 h, 6 h and 96 h) with three biological replicates for each time point. In total, eighteen samples were used for transcriptome sequencing. According to three time points, the samples were: A0, A1, A2, B0, B1 and B2 (Table 3).

**Table 3:** Samples for the transcriptome sequencing

Rootstock	Sample	Time of inoculation
Selfrooted	A0	0 h
	A1	6 h
	A2	96 h
*A.scoparia*	B0	0 h
	B1	6 h
	B2	96 h

### Transcriptome assembly and gene functional annotation

Transcriptome assembly was finished based on these high-quality reads of all samples by using Trinity software [69]. Raw reads were processed to remove reads which containing adaptors, reads of low quality (more than 50% bases with small Qphred ≤5), or with more than 10% ambiguous bases (N) to get clean reads for the following analysis. The longest transcript from all libraries was selected as the non-redundant unigene of this study.

To obtain gene functional annotations of *Chrysanthemum morifolium* T. ‘Hangbaiju’ transcriptome, the BLAST searches were performed using the sequences of the resulting unigenes based on the following databases: NCBI nonredundant nucleotide sequences (Nt), NCBI nonredundant protein sequences (Nr), Swiss-Prot, euKaryotic Ortholog Groups (KOG), GO (Gene Ontology), KEGG (Kyoto Encyclopedia of Genes and Genomes). The E-value threshold was set to 1E− 3 in the alignments to KOG. For the alignments to Swiss-Prot and Nt, the E-value threshold was 1E-5. KEGG Automatic Annotation Server (http://www.genome.jp/kegg/kaas/) was used for the KEGG annotations [77]. The GO annotations were performed with Blast 2 GO v2.5 [78] which according to the Nr and Pfam annotations. Pathway assignments were also mapped based on the KEGG database.

### Differential expression analysis

Fragments Per Kilobase per Million fragments (FPKM) method [79] was used to numerate the unigene expression level. FPKM values were used to demonstrate the gene expression levels in different samples. To examine the effects of the different aphid infestation times on the gene expression patterns of leaves in the Cm / Cm and Cm / As plants, the density distributions of FPKM values were compared among different aphid infestation time groups in this study. DEGs were determined based on an absolute value of fold change ≥2 and a threshold of q-value ≤0.05. Here, the q-value was adopted to adjust the *P*-values in multiple hypothesis tests [80]. In this study, hierarchical clustering was used to demonstrate the differential gene expression patterns between different aphid stress time points in the Cm / Cm and Cm / As plants. According to the functional annotations of the resulting unigenes, putative functions of the differentially expressed genes (DEGs) were deduced to discover the candidate genes for aphid infestation in chrysanthemum.

### Validation of RNA-Seq data by qRT-PCR

The expression of 15 DEGs in chrysanthemum was analyzed by qRT-PCR with three replicates to verify the RNA-Seq data. Prime Script™RT reagent qRT-PCR Kit with gDNA Eraser (Takara, Dalian, China), cDNA was synthesized, and genomic DNA was removed from total RNAs. The PCR mixture included 12.5 μL SYBR® Premix Ex Taq™II, 9.50 μL ddH_2_O, 1 μL of each gene-specific primer (10 μM) and 1 μL cDNA template. The qRT-PCR assays were performed with the following program: 94 °C for 3 min; 40 cycles of 94 °C for 20 s, 57 °C for 20 s and 72 °C for 30 s. Relative expression values of genes were calculated by the formula of 2 (38-value of Cp) [81]. Cp indicates the intersection point and the cycles during which the signals were higher than the threshold. If the value of Cp exceeded 38, the data had high uncertainty. The relative expression value of actin was used as an internal control. Three technical replicates were performed for all fifteen selected genes.

### Determination of physiological indexes of chrysanthemum during aphid infestation

Leaves of seedlings under aphid stress were harvested at 0 h, 6 h, 24 h, 48 h, 72 h, and 96 h to determine the physiological indexes. The activities of phenylalanine ammonia lyase (PAL) and catalase (CAT) were determined by the “Plant Physiology Experiment Instruction” [82], and the content of soluble sugar was determined by the “Plant Physiology Experimental Technique” [83]. The flavonoid content was determined according to Jia [84]. The activity of lipoxygenase (LOX) was measured as described by Zhang et al. [85]. Each determination included three biological and technical replicates.

### Statistical analyses

Statistical analyses of aphid numbers, enzymes and metabolites were performed by ANOVA (SAS Institute, Cary, NC, USA) using SPSS v. 21.0 (SPSS Inc., Chicago, IL, USA), and the determinations were expressed as the means ± standard error (SE) from a minimum of three replicates. Differences between means were compared using Tukey’s multiple range tests at *P* < 0.05 or *P* < 0.01.

## Supplementary information

**Additional file 1: Figure S1.** Composition of raw reads in the eighteen RNA libraries. **Figure S2.** Unigene Transcript length distribution. **Figure S3.** Functional classification and pathway assignment of DEGs by GO and KEGG.

**Additional file 2: Table S1.** Genes involved in sucrose metabolism to aphid infestation responses.

**Additional file 3: Table S2.** Genes involved in secondary metabolism to aphid infestation responses.

**Additional file 4: Table S3.** Genes involved in Plant hormone signaling pathway to aphid infestation responses.

**Additional file 5: Table S4.** Genes involved in down stream responses.

**Additional file 6: Table S5.** Genes involved in Plant-pathogen interaction to aphid infestation responses.

## Data Availability

The datasets used and analysed during the current study are available from the corresponding author on reasonable request.

## Abbreviations

BP: Biological process
CC: Cellular component
cDNA: Complementary DNA
CHS: Chalcone synthase gene
Ct: Threshold cycle
DEGs: Differentially expressed genes
FPKM: Fragments Per Kilobase per Million fragments
GO: Gene Ontology
KEGG: Kyoto Encyclopedia of Genes and Genomes
KO: KEGG ortholog database
KOG/COG: Clusters of orthologous groups of proteins
MF: Molecular function
NR: NCBI non-redundant protein sequences
NT: NCBI non-redundant nucleotide sequences
Pfam: Protein family
qRT-PCR: Quantitative real-time-polymerase chain reaction
RNA-Seq: High-throughput RNA-sequencing
Swiss-Prot: A manually annotated and reviewed protein sequence database
