# Supplementary material for: Comprehensive transcriptome analysis of grafting onto Artemisia scoparia W. to affect the aphid resistance of chrysanthemum (Chrysanthemum morifolium T.)
Source: BMC Genomics. 2019 Oct 25;20:776. doi: 10.1186/s12864-019-6158-3 (PMC6815057; doi:10.1186/s12864-019-6158-3)
Supplement: Supplementary file 1 — Additional file 1: Figure S1. Composition of raw reads in the eighteen RNA libraries. Figure S2. Unigene Transcript length distribution. Figure S3. Functional classification and pathway assignment of DEGs by GO and KEGG. [file 12864_2019_6158_MOESM1_ESM.docx]

**Comprehensive transcriptome analysis of grafting onto** ***Artemisia scoparia* W. to affect the aphid resistance of chrysanthemum (*Chrysanthemum morifolium* T. )**

Xue-ying Zhang, Xian-zhi Sun*, Sheng Zhang, Jing-hui Yang, Fang-fang Liu, Jie Fan

College of Horticulture, Shandong Agricultural University, 61 Daizong Street, Taian 271018, China

*Corresponding author; E-mail: [sunxianzhi@126.com](mailto:sunxianzhi@126.com)

**Additional file 1**

**
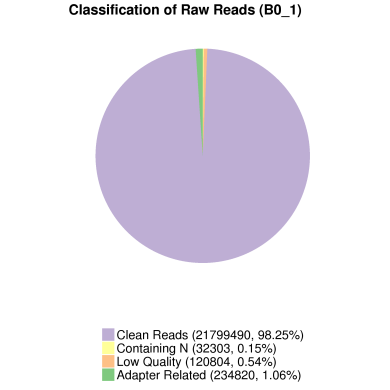

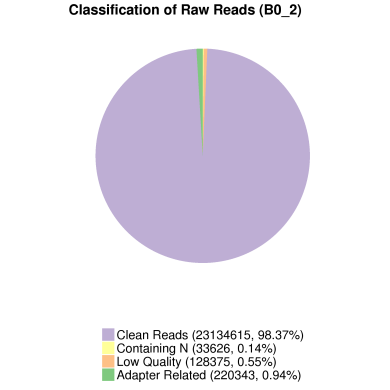

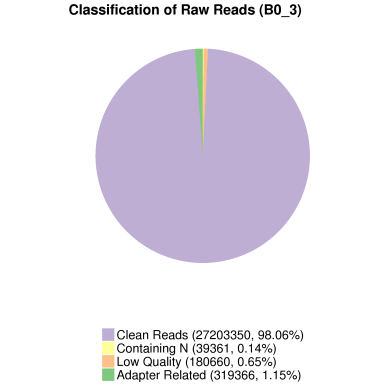
**

**
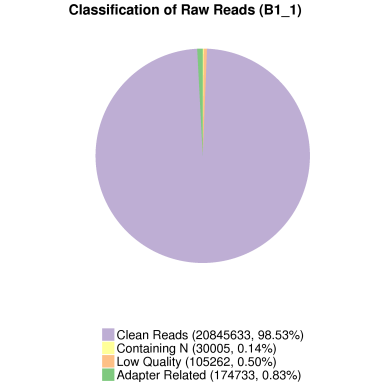

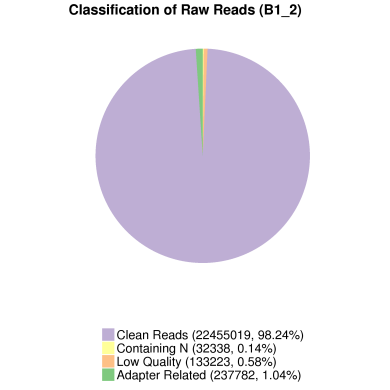

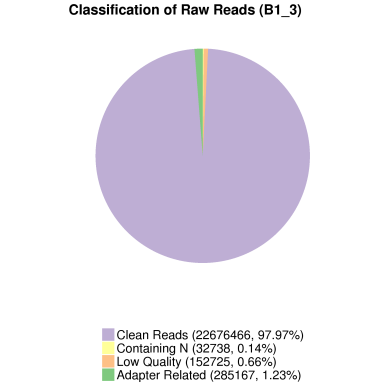
**

**
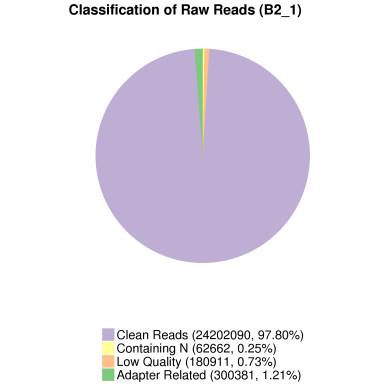

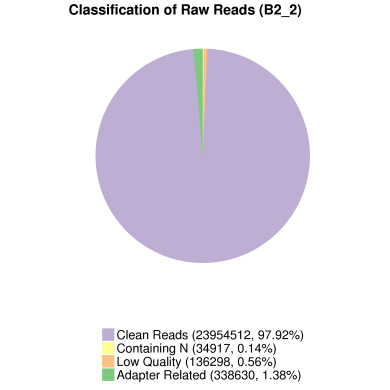

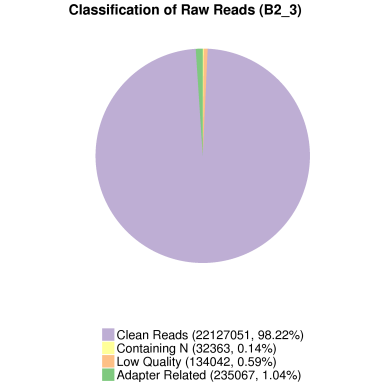

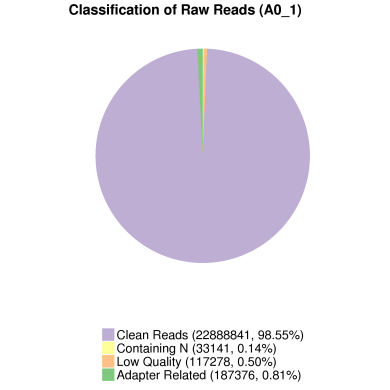

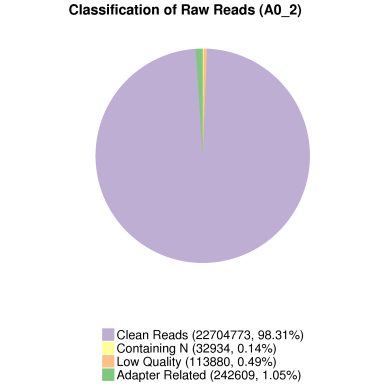

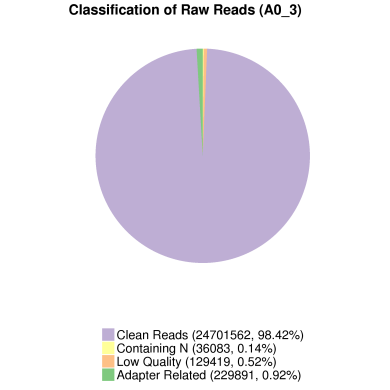
**

**
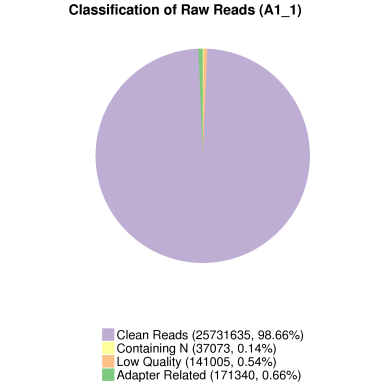

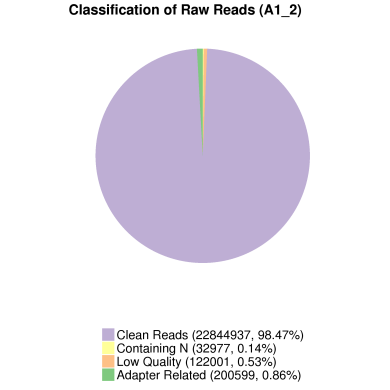

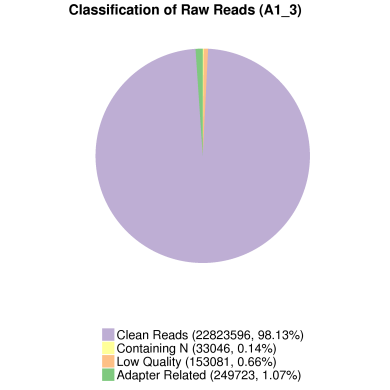
**

**
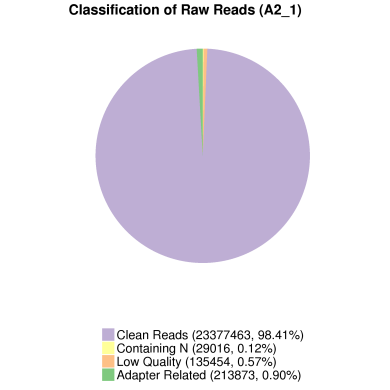

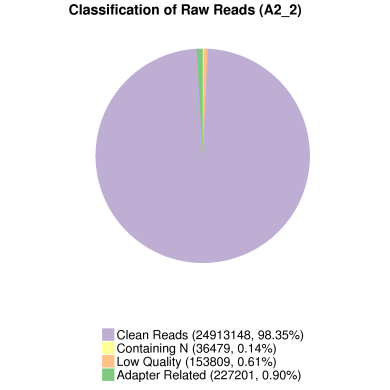

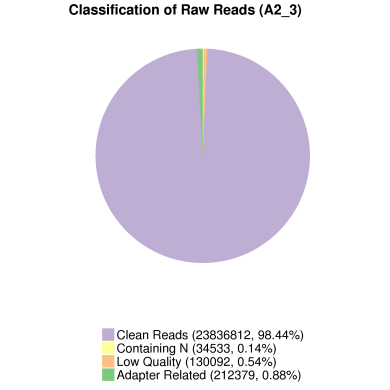
**

**Figure S1. Composition of raw reads in the eighteen RNA libraries.**


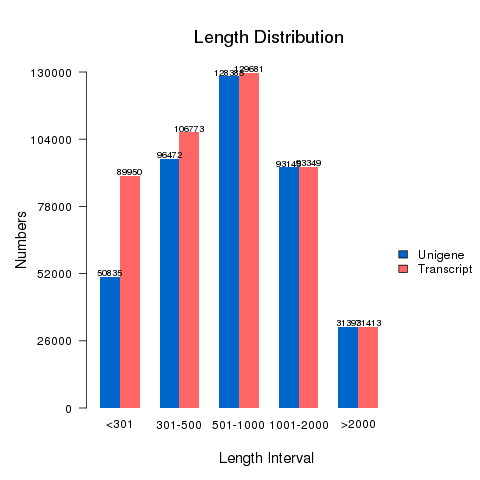


**Figure S2. Unigene Transcript length distribution**


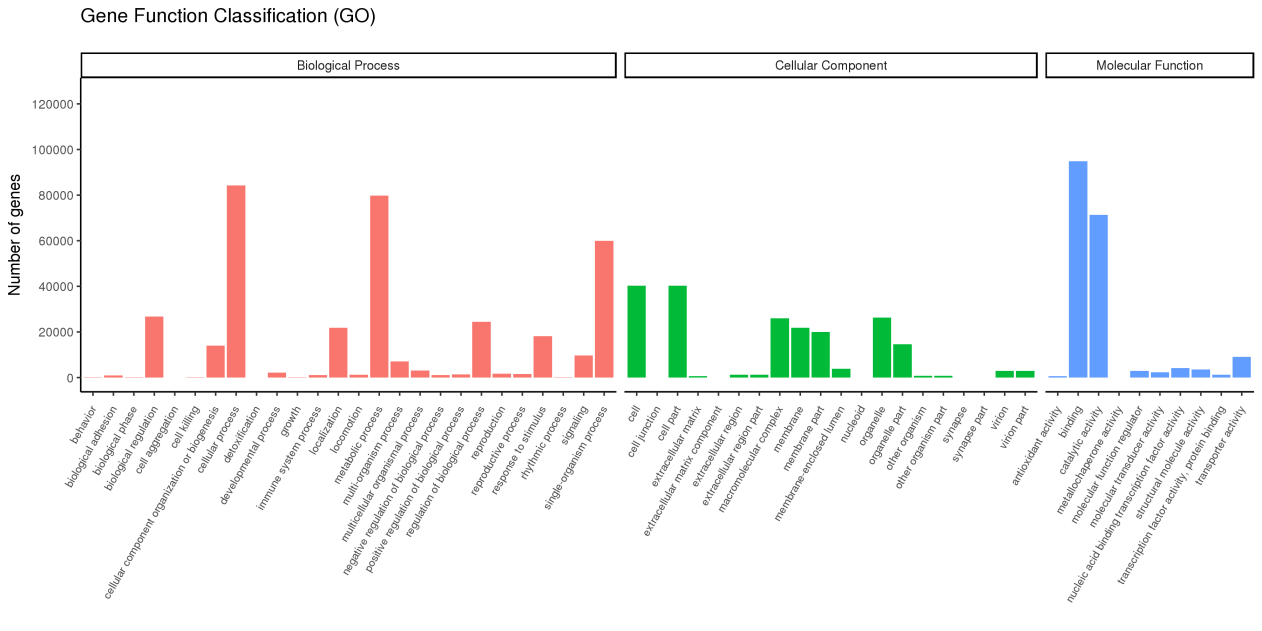


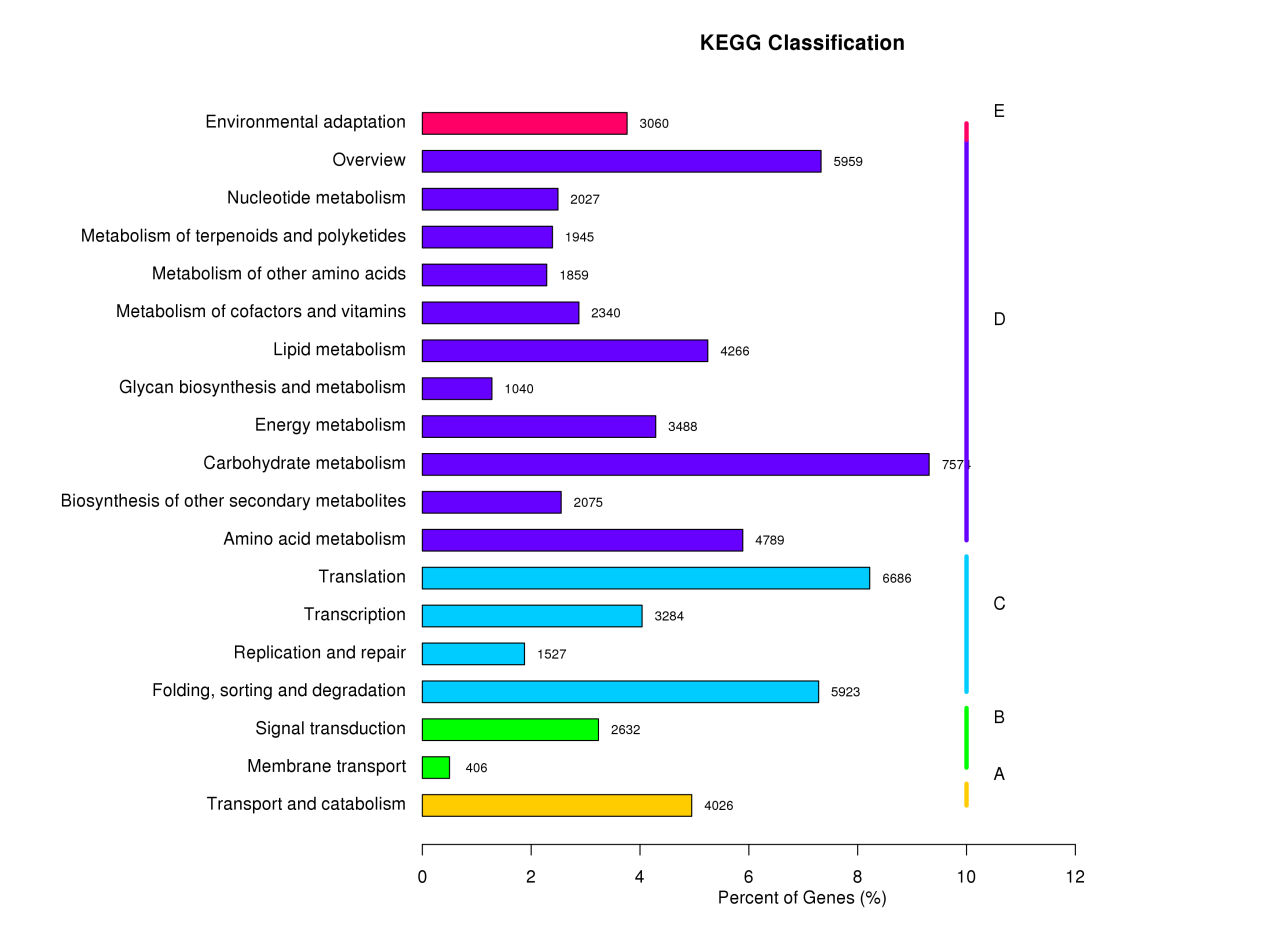


**Figure S3. Functional classification and pathway assignment of DEGs by GO and KOG.**
